# Molecular mechanisms of tiling and self-avoidance in neural development

**DOI:** 10.1186/1756-6606-3-28

**Published:** 2010-10-11

**Authors:** Scott Cameron, Yong Rao

**Affiliations:** 1 McGill Centre for Research in Neuroscience, McGill University Health Centre, 1650 Cedar Avenue, Montreal, Quebec H3G 1A4, Canada; 2 Department of Neurology and Neurosurgery, McGill University Health Centre, 1650 Cedar Avenue, Montreal, Quebec H3G 1A4, Canada; 3 Department of Medicine, McGill University Health Centre, 1650 Cedar Avenue, Montreal, Quebec H3G 1A4, Canada

## Abstract

Recent studies have begun to unravel the molecular basis of tiling and self-avoidance, two important cellular mechanisms that shape neuronal circuitry during development in both invertebrates and vertebrates. Dscams and Turtle (Tutl), two Ig superfamily proteins, have been shown to mediate contact-dependent homotypic interactions in tiling and self-avoidance. By contrast, the Activin pathway regulates axonal tiling in a contact-independent manner. These cell surface signals may directly or indirectly regulate the activity of the Tricornered kinase pathway and/or other intracellular signaling pathways to prevent the overlap between same-type neuronal arbors in the sensory or synaptic input field.

## 

Neuronal circuit formation requires proper interactions between neurites of developing neurons during embryonic development. A neurite can interact with another neurite in a homotypic (i.e. interaction between neurites from same-type neurons or sister branches from the same neuron) or heterotypic manner (i.e. interaction between neurites from different types of neurons). These interactions have been shown to play important roles in regulating a variety of processes such as axonal guidance, dendrite morphogenesis and synaptogenesis [1-3]. This review focuses on molecular details of homotypic interactions underlying tiling and self-avoidance, two important cellular mechanisms that pattern neuronal circuitry in the nervous system.

Tiling involves the recognition between certain same-type or functionally equivalent neurons, which allows the neurites from same-type neurons to completely cover the sensory or synaptic input field with no or minimal overlap. Tiling is likely required for providing an anatomical basis for parallel detection of same-type sensory information in the receptive field and thus allows the spatial discrimination of sensory information. The phenomenon of tiling was first discovered in the cat retina by Boycott and colleagues in 1981 [4] (Fig. 1A). Their work demonstrates that certain same-type retinal ganglion neurons (i.e. ON-brisk-transient cells and OFF-brisk-transient cells) are organized in a mosaic pattern in the retina, and so the receptive field is covered completely but non-redundantly with each subtype of α-ganglion neurons. Later studies showed that many of ~50 types of mammalian retinal neurons displayed some degree of tiling pattern, which appears to be essential for unambiguously processing visual information from the external world [5]. Tiling has also been observed in the Drosophila visual system [6] (Fig. 1B), and many other neuronal systems in both vertebrates and invertebrates [4,7-13].

**Figure 1:**
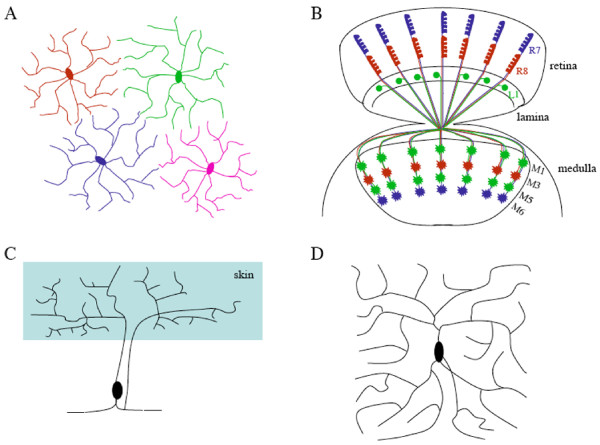
**Examples of tiling and self-avoidance in vertebrates and invertebrates**. A, A simplified diagram showing the tiling of vertebrate retinal ganglion neurons, based on results in [4]. B, Axonal tiling contributes to the organized columnar projection pattern of R7 and R8 photoreceptor neurons and L1 lamina neurons in the medulla of the Drosophila visual system. While L1 neurons arborize at both M1 and M5 sub-layers, R7 and R8 axons terminate at M6 and M3 sub-layers, respectively. Genetic dissection of neuronal circuit formation in the fly visual system has contributed significantly to our understanding of neuronal positioning, axon guidance and neuronal target selection (e.g. [53-57]). C, A schematic diagram showing the non-overlapping coverage of the receptive field by sister branches from a Pv mechanosensory neuron in leech, based on results in [14]. D, A simplified diagram showing self-avoidance in a Drosophila class IV da neuron, where sister branches tend to avoid each other.

Homotypic interactions can also regulate self-avoidance where sister neurites from the same neuron repel each other to ensure the uniform coverage of receptive fields for effectively processing sensory information. The concept of self-avoidance was first proposed from studies in the giant Amazon leech in the early 1980s [14,15] (Fig. 1C). Those studies demonstrated that sister arborizing neurites of the same mechanosensory neuron did not overlap, which is likely required for maximizing the capability to sense mechanical stimulation in the body wall. Self-avoidance has been identified in a number of different neuronal cell types in both vertebrates and invertebrates [9,10,16-19] (Fig. 1D).

## Contact-dependent mechanism: homotypic interaction mediated by cell surface recognition molecules in tiling and self-avoidance

It has long been proposed that specific contact-dependent cell surface recognition mechanisms must exist to allow a neurite to repel a like neurite but not a unlike neurite in tiling and self-avoidance. For instance, it was shown that removal of a ganglion neuron in the rat retina led to the invasion of dendrites from its neighboring same-type neuron into the vacant area [12]. Similarly, surgical removal of an axonal branch of a mechanosensory neuron in leech caused the overgrowth of sister branches in the receptive field [15]. Live time-lapse analysis of developing dendrite arborization (da) sensory neurons in Drosophila, and horizontal retinal cells in mice, show that neurites of those neurons often touch and then retract when they come into contact with neurites of the same type [20-23], suggesting an active role for homotypic repulsion in tiling and self-avoidance. Recent studies have begun to define the molecular basis of contact-dependent homotypic interactions, leading to the identification of immunoglobulin superfamily proteins Dscam and Turtle (Tutl), two important molecular determinants of tiling and self-avoidance.

### Dscams in tiling and self-avoidance

**Figure 2:**
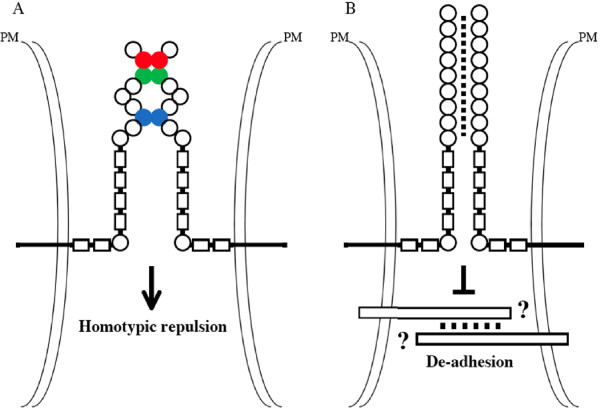
**Proposed models for the action of Drosophila and vertebrate Dscams**. A, Drosophila Dscam1 mediates homotypic repulsion in tiling and self-avoidance. The homophilic binding specificity of Dscam1 is determined by its Ig2 (red), Ig3 (green) and Ig7 (purple) domains. Half of Ig2, half of Ig3, and entire Ig7 domains are encoded by one of 12, 48, and 33 alternative exons, respectively. Thus, the extensive alternative splicing can generate 19,008 Dscam1 isoforms with different binding specificity. The binding between two Dscam isoforms only occurs if all three Ig domains (i.e. Ig2, Ig3 and Ig7) are identical. B, Vertebrate Dscams may mediate de-adhesion in tiling and self-avoidance by down-regulating the function of some unknown cell-type-specific adhesion molecules. The binding region on vertebrate Dscams is unknown, and presumably contains one or more of Ig domains. Circles, Ig domains. Rectangles, fibronectin type-III repeats. PM, plasma membrane

The first clue for a cell surface recognition mechanism underlying tiling and self-avoidance came from recent studies on the Down's syndrome cell adhesion molecule Dscam [24]. Dscam belongs to the immunoglobulin superfamily, and consists of ten Ig-like domains, six fibronectin type-III repeats, a singe transmembrane region and a cytoplasmic domain [25] (Fig. 2). There are two Dscam genes (i.e. Dscam and Dscam-like or DscamL1) in vertebrates [26], and four Dscam genes (Dscam 1-4) in Drosophila melanogaster [27,28]. Both invertebrate and vertebrate Dscams mediate homophilic binding [29-31] (Fig. 2). In Drosophila, the Dscam1 gene has the potential to generate a large number of isoforms (i.e. 38,016) that possess 19,008 different binding specificities through alternative splicing [27]. *In vitro *studies demonstrate that each isoform binds to itself but shows little binding to a different isoform [29,32] (Fig. 2).

The function of Dscams in self-avoidance was first suggested by genetic analysis of Dscam1 in the Drosophila olfactory system [33,34]. Axons of neurons in the mushroom body normally bifurcate to generate two sister branches that innervate two distinct targets. The work by Lee and colleagues provided evidence that Dscam is required for the segregation of sister branches in mushroom body neurons [33]. Later studies by Zipursky and colleagues revealed that different subtype of mushroom body neurons randomly express a different set of Dscam1 isoforms [34]. However, within a single mushroom body neuron, sister branches express the identical set of Dscam1 isoforms, which mediates homotypic repulsion for segregation of sister branches [35]. Branches from different MB neurons overlap due to their difference in the expression of Dscam1 isoforms. A more recent study by Zipursky and colleagues provides further evidence that the wiring of ~2,500 neurons in the mushroom body requires at least 4,752 potential Dscam1 isoforms for distinguishing self from non-self in the projection of sister branches [36].

Dscam1 has also been shown to be required for dendritic self-avoidance by three independent studies [37-39]. Loss of Dscam1 led to severe self-avoidance defects in Drosophila dendrite arborization (da) sensory neurons as abnormal fusion between sister branches was frequently observed in all four classes of da neurons. That expression of a single Dscam1 isoform could rescue self-avoidance defects supports the idea that homotypic repulsion mediated by the binding of identical Dscam1 isoforms on opposing sister branches controls dendritic self-avoidance in da neurons. Conversely, co-existence of branches from different classes of da neurons in the sensory field results likely from differential expression of Dscam1 isoforms, which was shown in experiments that overexpressing a single Dscam1 isoform forced the segregation of branches from different-type da neurons [37-39]. Like that in the mushroom body, it was shown that at least thousands of Dscam1 isoforms are required for da neurons to distinguish self from non-self branches [36]. The signaling mechanism by which Dscam1 mediates homotypic repulsion remains unknown. Since deletion of the Dscam1 cytoplasmic domain prevented Dscam-mediated repulsion without affecting its homophilic binding activity [37], it appears likely that the conversion of Dscam1-mediated homophilic binding into homotypic repulsion requires the interaction between its cytoplasmic domain and downstream signaling proteins. The molecular identity of downstream signaling proteins, however, remains unknown.

The first experimental evidence for the role of Dscams in tiling came from the study of the second Dscam gene Dscam2 in the Drosophila visual system [28]. The medulla of the Drosophila visual system consists of ~800 regularly spaced columns, each contains axons from R7 and R8 photoreceptor neurons and L1-L5 lamina neurons that process different visual information (reviewed by [40,41]) (Fig. 1B). Axonal terminals in one column do not overlap with same-type terminals in an adjacent column, which is necessary for the discrimination of visual information from different points in space. Genetic mosaic analysis indicates that Dscam2 functions both cell-autonomously and cell non-autonomously in the tiling of L1 lamina neurons [28]. It was shown that a Dscam2 mutant axonal terminal frequently invades its neighboring columns, while a column containing a Dscam2 mutant L1 axon was also frequently invaded by a wild-type L1 axon in neighboring columns. Consistently, biochemical and cell biology studies show that Dscam2, like Dscam1, also mediates homophilic binding. Thus, like the action of Dscam1 in self-avoidance, Dscam2 may mediate homotypic repulsion in the tiling of L1 neurons in the Drosophila visual system.

Recent studies have also implicated a role for Dscams in tiling and self-avoidance in the vertebrate visual system [42,43]. Loss of mouse Dscam disrupted the regular mosaic pattern of retinal ganglion cells and amacrine cells, and caused the hyper-fasciculation of neurites [43]. While loss of mouse Dscaml1 affected the tiling and self-avoidance of amacrine cells and rod bipolar cells [42]. The action of vertebrate Dscams, however, appears different from that of Drosophila Dscam1. First, vertebrate Dscam genes do not undergo extensive alternative splicing, and are thus unlikely to function as a molecular code for self recognition of many different types of retinal neurons. Second, both Dscam and Dscaml1 are widely expressed in the mouse retina. While Dscam is expressed in most types of retinal ganglion neurons and some types of amacrine cells, Dscaml1 is present on most types of amacrine cells and rod bipolar cells. And third, in Dscam or Dscaml1 mutant retina, only same-type neurites/cells abnormally sticked together, arguing against a role for vertebrate Dscams to encode cell-type identity. Thus, unlike the action of Drosophila Dscam1 in homotypic repulsion (Fig. 2A), it appears more likely that vertebrate Dscams mediate de-adhesion and mask certain cell-type-specific adhesive cues, thus preventing the fusion of same-type neurites to establish tiling and self-avoidance (Fig. 2B). Alternatively, vertebrate Dscams may form a complex with cell-type-specific adhesion molecules, and thus converts cell-type-specific adhesion into cell-type-specific repulsion.

### Turtle/Dasm1/IgSF9 in tiling and self-avoidance

**Figure 3:**
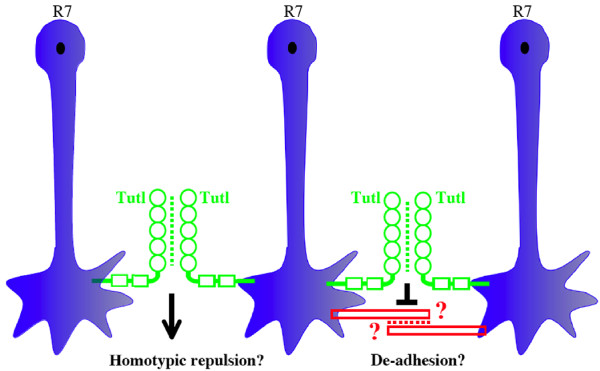
**Proposed models for the action of Tutl**. The homophilic binding between two Tutl proteins on opposing cell surface may be mediated by one or more of its Ig domains. Tutl may mediate homotypic repulsion. Alternatively, Tutl may mediate de-adhesion by antagonizing the function of certain R7-specific cell adhesion molecules. Circles, Ig domains; Rectangles, fibronectin type-III repeats.

The Drosophila Turtle (Tutl) protein is the second cell surface determinant shown to regulate homotypic interaction in tiling and self-avoidance [44,45]. Tutl is a member of the evolutionarily conserved Turtle/Dasm1/IgSF9 subfamily of the Ig-superfamily, which share a common architecture comprising of an extracellular region containing five Ig domains and two fibronectin type-III repeats, a single transmembrane region, and a cytoplasmic domain [46-48] (Fig. 3).

Our recent study demonstrates a role for Tutl in tiling R7 photoreceptor axons in Drosophila [44]. In the Drosophila visual system, R7 axons are segregated into regularly spaced columns where they establish synaptic connections at the M6 sub-layer of the medulla (Fig. 1B). In *tutl *mutants, R7 terminals frequently extended laterally and fused with other R7 terminals in neighboring columns. Single cell mosaic analysis indicates that *tutl *is required both cell-autonomously and cell non-autonomously for preventing the fusion between neighboring R7 terminals. This result, together with that Tutl mediates homophilic cell-cell aggregation in cultured cells [44], support the idea that the homophilic binding of Tutl on adjacent R7 terminals regulates contact-dependent homotypic interactions in R7 tiling. Consistently, we showed that genetic ablation of neighboring R7 terminals significantly decreased the frequency of a *tutl *mutant R7 axon to invade its neighbors. We propose two alternative models for the action of Tutl in R7 tiling (Fig. 3). In the first model, Tutl may act like Dscam1 to mediate homotypic repulsion, thus preventing the fusion between neighboring R7 terminals. Alternatively or additionally, Tutl may function like vertebrate Dscams to mediate de-adhesion by antagonizing the function of certain cell adhesion molecules on R7 terminals.

Tutl has also been implicated a role in regulating dendritic self-avoidance [45]. Loss of *tutl *significantly increased the overlap between sister branches in class IV da neurons. However, unlike Dscam1 that is required in all four classes of da neurons [37-39], *tutl *mutations did not affect self-avoidance in class I, II and III da neurons [45]. One possible explanation is that in the absence of Tutl, the expression of Dscam1 in class I-III da neurons is sufficient for self-avoidance, while both Dscam1 and Tutl are required in class IV da neurons to ensure the non-overlapping distribution of sister branches. Like Dscam1 [37-39], Tutl is not required for tiling da neurons [45].

## Contact-independent mechanism: control of intrinsic terminal growth by the Activin pathway in axonal tiling

**Figure 4:**
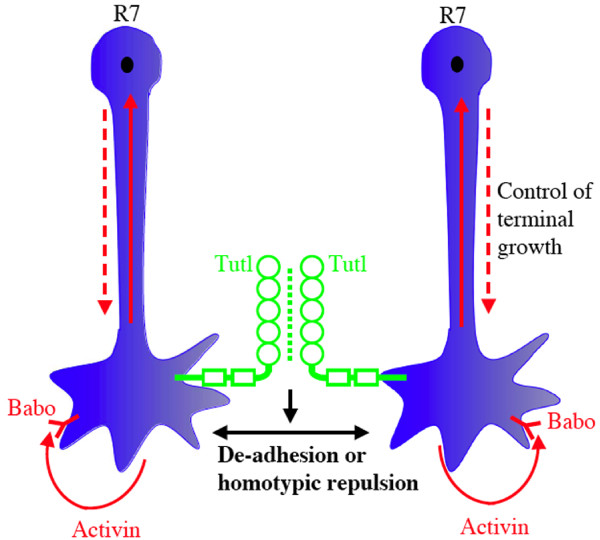
**Co-operation between the Activin pathway and Tutl in the tiling of Drosophila R7 photoreceptor axons**. Activin functions as an autocrine signal to activate its receptor Babo on R7 terminals, which in turn induces the phosphorylation of Smad2. The phosphorylated Smad2 is then transported by Importin α3 into the nucleus to regulate the expression of some unknown target genes, which directly control R7 terminal growth. This Activin-mediated intrinsic growth control functions together with Tutl-mediated homotypic interaction to regulate R7 tiling. Circles, Ig domains; Rectangles, fibronectin type-III repeats.

While it is well established that contact-dependent homotypic repulsion plays a key role in axonal/dendritic tiling, several studies indicate that tiling also involves a contact-independent mechanism [6,19]. For instance, it was shown that in Brn3b-/- and Math5-/- mice, although retinal ganglion neurons do not contact each other throughout development due to the great reduction in the number of ganglion neurons, they nevertheless displayed a regular mosaic pattern and normal dendritic morphology [19]. Similarly, Ashley and Katz reported that in the Drosophila visual system, R7 photoreceptor axons were still restricted to their own columns even when their neighboring R7 neurons were removed by genetic manipulation [6]. These studies indicate the existence of an intrinsic mechanism restricting the growth of neuronal arbors in the absence of neighboring same-type neurons. However, it appears clear that when same-type neighbors are present, this intrinsic growth control has to function together with a contact-dependent tiling mechanism to overcome the high affinity between same-type neurites due to the presence of homophilic cell adhesion molecules (e.g. NCAM in vertebrate retinal neurons and N-cadherin in Drosophila R7 photoreceptors). While the nature of the intrinsic mechanism in the vertebrate visual system remains unknown, a recent study by Lee and colleagues provides convincing evidence that in the Drosophila visual system, the Activin signaling pathway contributes to the contact-independent tiling mechanism [49] (Fig. 4).

Genetic studies show that mutations in the genes encoding components of the Activin pathway caused specific defects in R7 tiling as R7 terminals frequently extended laterally to invade their neighboring columns [49]. Those components include dActivin, the fly Activin receptor Baboon (Babo), the downstream effector dSmad2 or nuclear transporter Importin-α3. Unlike Tutl that acts both cell-autonomously and cell non-autonomously [44], the Activin pathway is only required cell autonomously in R7 tiling [49]. The frequency of tiling error for a mutant R7 axon defective in Activin signaling could be significantly increased when its neighboring R7 axons were ablated by genetic manipulation, suggesting that the Activin pathway is required for controlling intrinsic terminal growth but not homotypic repulsion. Our recent genetic analysis showed that reducing the dosage of *tutl *could significantly enhanced the tiling phenotype in mutants defective in Activin signaling [44], suggests strongly that the Activin-mediated intrinsic growth control functions together with homotypic repulsion (or de-adhesion) mediated by Tutl to regulate R7 tiling (Fig. 4).

## Intracellular signaling mechanism: the action of the Tricornered kinase pathway

**Figure 5:**
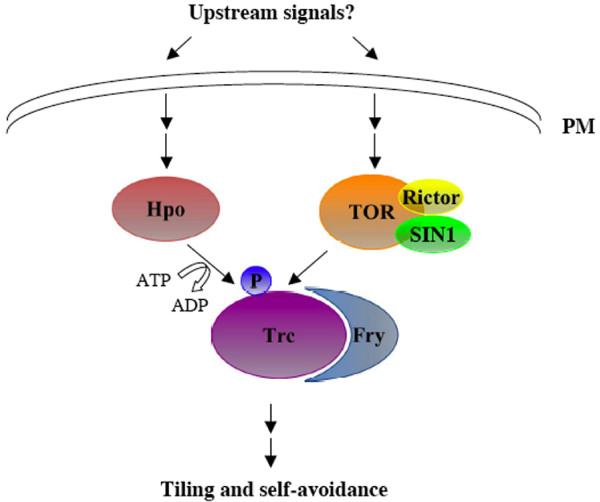
**The function of the Trc signaling pathway in tiling and self-avoidance**. Unidentified cell surface signals activate Hpo and TOR, which in turn up-regulate the activity of the Trc and Fry complex. Trc may then modulate the activity of certain cytoskeletal regulators to control the growth of neuronal arbors. PM, plasma membrane.

Recent genetic dissection of dendritic patterning in Drosophila da neurons has led to the identification of several components of the Tricornerd kinase pathway in dendritic tiling and self-avoidance (Fig. 5), including the Tricornered (Trc) Ser/Thr kinase [22], its activator Furry (Fry) [22], upstream regulators such as Ste20-like Ser/Thr kinase Hippo (Hpo) [50] and the target of rapamycin (TOR) Ser/Thr kinase [51].

Trc belongs to the NDR family of Ser/Thr kinases [52]. In Drosophila, loss of Trc or its activator Fry increased the crossings between sister branches from the same class IV da neuron and also increased the overlap between dendritic fields of two adjacent class IV da neurons, indicating a role for Trc and Fry in both tiling and self-avoidance [22]. The function of Trc and Fry in tiling and self-avoidance appears to be evolutionarily conserved as the C. elegans Trc (Sax-1) and Fry (Sax-2) have been shown to function similarly in mechano-sensory neurons [8]. Biochemical and genetic studies indicate that the function of Trc in tiling and self-avoidance is regulated by two Ser/Thr kinases Hpo and TOR [50]. Hpo activates Trc by directly phosporylating Trc at Thr 449 [50], whereas the target of Rapamycin complex 2 (TORC2) consisting of the TOR kinase and its co-factors Sin1 and Rictor associates with Trc and may up-regulate its activity in a different way [51].

The mechanism by which the Trc signaling pathway is activated by cell surface signals in tiling and self-avoidance remains elusive. While a similar self-avoidance phenotype in class IV da neurons was observed in mutants defective in Dscam1 or Tutl [22,37-39,45], no physical or genetic interaction has been detected between *Dscam1 *and *Trc*, or between *tutl *and *Trc*. In addition, unlike Dscam1 and Tutl, Trc is also required for tiling class IV da neurons [22], indicating that at least in class IV da neurons the function of the Trc pathway in tiling is regulated by cell surface signals other than Dscam1 and Tutl. Those cell surface signals may activate the Trc pathway to promote contact-dependent repulsion between same-type neurites in tiling and self-avoidance. Alternatively or additionally, they may activate the Trc pathway to control the terminal growth of neuronal arbors in a contact-independent manner.

## Concluding remarks

Recent studies have significantly advanced our understanding of the molecular mechanism of tiling and self-avoidance in the developing nervous system. The identification of Dscams and Turtle sheds light on the molecular basis of contact-dependent homotypic interactions underlying tiling and self-avoidance. While the Drosophila Dscam1 functions as a self-recognition code to mediate homotypic repulsion, mouse Dscams appear to act as a general de-adhesion factor that may antagonize the function of certain cell-type-specific adhesion molecules. Whether Turtle mediates homotypic repulsion or de-adhesion remains to be determined. Unlike Dscams and Turtle, the Activin pathway regulates the tiling of Drosophila R7 photoreceptor axons in a contact-independent manner. These cell surface signals may directly or indirectly regulate the activity of the Tricornered kinase pathway and/or other intracellular signaling pathways. Future studies will be necessary to determine the exact mechanism by which the signals from the activation of these cell surface receptors are converted into the changes in the motility of axonal/dendritic terminals in tiling and self-avoidance.

## Competing interests

The authors declare that they have no competing interests.

## Authors' contributions

SC and YR wrote the manuscript. All authors read and approve the manuscript.
